# Cyclase-associated protein interacts with actin filament barbed ends to promote depolymerization and formin displacement

**DOI:** 10.1016/j.jbc.2023.105367

**Published:** 2023-10-19

**Authors:** Nikita Alimov, Gregory J. Hoeprich, Shae B. Padrick, Bruce L. Goode

**Affiliations:** 1 Department of Biology, Rosenstiel Basic Medical Science Research Center, Brandeis University, Waltham, Massachusetts, USA; 2 Department of Biochemistry and Molecular Biology, Drexel University, Philadelphia, Pennsylvania, USA

**Keywords:** cyclase-associated protein, actin, turnover, cofilin, ADF, TIRF microscopy

## Abstract

Cyclase-associated protein (CAP) has emerged as a central player in cellular actin turnover, but its molecular mechanisms of action are not yet fully understood. Recent studies revealed that the N terminus of CAP interacts with the pointed ends of actin filaments to accelerate depolymerization in conjunction with cofilin. Here, we use *in vitro* microfluidics-assisted TIRF microscopy to show that the C terminus of CAP promotes depolymerization at the opposite (barbed) ends of actin filaments. In the absence of actin monomers, full-length mouse CAP1 and C-terminal halves of CAP1 (C-CAP1) and CAP2 (C-CAP2) accelerate barbed end depolymerization. Using mutagenesis and structural modeling, we show that these activities are mediated by the WH2 and CARP domains of CAP. In addition, we observe that CAP collaborates with profilin to accelerate barbed end depolymerization and that these effects depend on their direct interaction, providing the first known example of CAP-profilin collaborative effects in regulating actin. In the presence of actin monomers, CAP1 attenuates barbed end growth and promotes formin dissociation. Overall, these findings demonstrate that CAP uses distinct domains and mechanisms to interact with opposite ends of actin filaments and drive turnover. Further, they contribute to the emerging view of actin barbed ends as sites of dynamic molecular regulation, where numerous proteins compete and cooperate with each other to tune polymer dynamics, similar to the rich complexity seen at microtubule ends.

The ability of cells to change shape and crawl depends on dynamic rearrangements of their actin cytoskeletons (1). The actin structures found in cells have distinct sizes and filamentous architectures, which must not only be assembled with spatiotemporal precision but also maintained in a state of flux (or subunit turnover) to retain their plasticity and provide sustained force for membrane remodeling (2, 3, 4). Although rapid actin network turnover is required for many actin-based processes *in vivo*, the molecular mechanisms that underlie actin filament turnover are still coming into focus (5, 6).

The process by which cells promote actin turnover can be deconstructed into four steps: (i) attenuation of barbed end growth, (ii) debranching and severing, (iii) dissociation of subunits from filament ends (*i.e.*, depolymerization), and (iv) monomer recycling (7). The efficiency of the first step (attenuation of barbed end growth) depends on whether or not barbed ends are bound to elongation-promoting factors such as formins and Ena/VASP and on the availability of factors promote displacement of elongators and/or cap barbed ends (8, 9, 10, 11, 12). Filament severing is achieved by cofilin working in concert with its cofactors AIP1, cyclase-associated protein (Srv2/CAP), coronin, and MICAL (13, 14, 15, 16, 17, 18, 19, 20, 21). Importantly, severing also can be antagonized by filament side-binding proteins such as tropomyosins (22, 23, 24, 25). However, even after a filament is severed into smaller fragments, the rate-limiting step in its disassembly is depolymerization, that is, the dissociation of subunits from filament ends. Mechanisms that increase the depolymerization rate at either the barbed or the pointed end can play an important role in driving actin network turnover. However, it is interesting that actin filaments inherently depolymerize much faster at their barbed ends than at their pointed ends (26, 27, 28).

Historically, the spotlight for filamentous actin (F-actin) disassembly and turnover has been focused at the pointed end (1, 29). The barbed end has been largely overlooked, presumably because it is the established site of filament growth in cells. Further, the cytosol of eukaryotic cells typically contains at least 5 to 10 μM of assembly-competent actin monomers (30) which is about two orders of magnitude higher than the critical concentration for assembly. Therefore, under cytosolic conditions, it was initially assumed that free barbed ends would only experience growth. However, recent studies have begun to call this view into question and are expanding our understanding of barbed end regulation (8, 10, 11, 31, 32, 33, 34, 35, 36). It is now apparent that a large number of different proteins dynamically associate and exchange with each other at filament barbed ends, competing and cooperating to control polymer dynamics. These barbed end–associated factors have diverse effects ranging from accelerated growth to decelerated growth to transient capping and depolymerization. Moreover, *in vivo* studies at the leading edge of cells suggest that disassembly may occur at barbed ends of filaments shortly after their assembly (5, 37, 38, 39, 40). Together, these observations identify the barbed end as a site that is important not only for actin network growth but also network turnover or disassembly.

One of the central players in actin turnover is cyclase-associated protein (CAP), a 57 kDa multi-domain protein that is conserved across the animal, fungal, and plant kingdoms (41, 42). Across diverse model organisms, genetic loss of CAP leads to severe defects in actin organization and actin-dependent cellular processes, including cell morphogenesis, cell motility, cell adhesion, and endocytosis (41, 42). In mammals, there are two CAP genes. CAP1 is ubiquitously expressed, while CAP2 is expressed primarily in muscle, neurons, and skin (43). A CAP1 knockout is embryonic lethal in mice (44), while a CAP2 knockout results in disorganized sarcomeres and cardiomyopathies (45). Furthermore, knocking down CAP1 in cells leads to pronounced defects in F-actin organization, cell motility, endocytosis, oogenesis, and neuronal growth cone dynamics (43, 46, 47, 48). Together, these observations demonstrate that CAP1 and CAP2 play a variety of crucial roles in actin regulation *in vivo*.

CAP has multiple biochemical effects on actin dynamics, and CAP directly interacts with F-actin, globular actin (G-actin), and other actin regulatory proteins such as profilin and cofilin. The N-terminal half of CAP (N-CAP) consists of an oligomerization domain and a helical folded domain (HFD) (Fig. 1*A*) and homo-oligomerizes to form hexamers and possibly tetramers (18, 49, 50, 51, 52, 53). CAP oligomers use their HFD domains to bind transiently to the sides of filaments (dwell time <1 s) and enhance cofilin-mediated severing (18, 50). Additionally, yeast and mammalian N-CAP fragments bind to the pointed ends of filaments (dwell time ∼2 s) and alone increase the rate of pointed end depolymerization by ∼4- to 7-fold (54, 55). Moreover, N-CAP synergizes with cofilin to promote pointed-end depolymerization at a rate of ∼50 subunits s^−1^ (>300-fold faster than control reactions).

**Figure 1:**
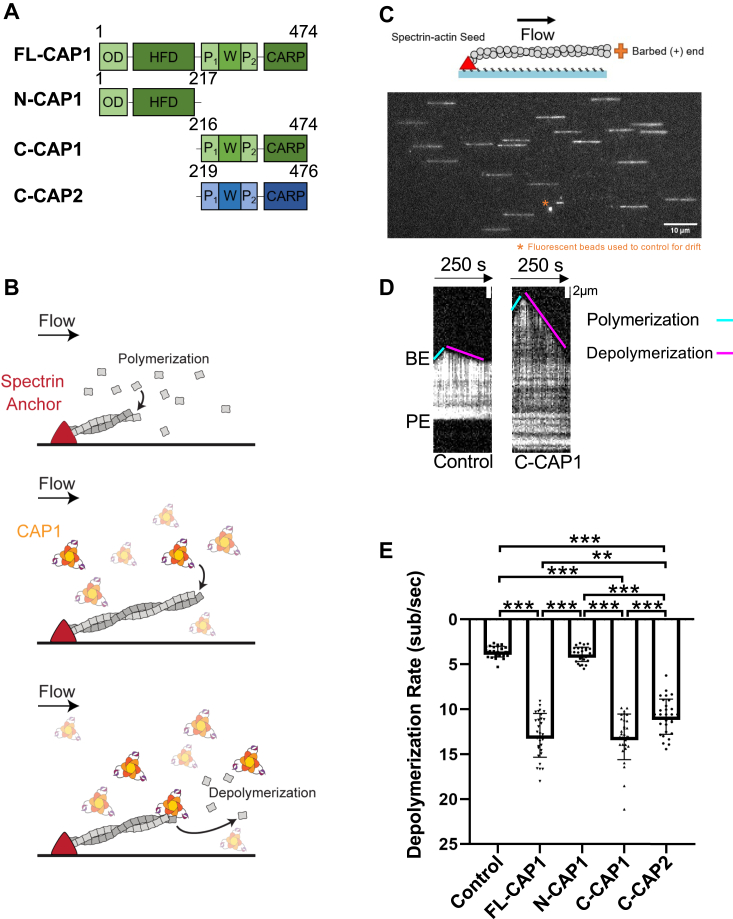
**CAP1 and CAP2 accelerate depolymerization at the barbed ends of actin filaments.***A*, domain layouts for mouse CAP1 and CAP2 polypeptides used in this study. *B*, experimental set up of microfluidics-assisted TIRF (mf-TIRF) assays. Alexa-488–labeled actin filaments were assembled from passively absorbed spectrin-actin seeds, resulting in filaments with pointed ends anchored on the *left* and free barbed ends growing on the *right*. After filaments were polymerized to 5 to 15 μm in length, then free actin monomers were washed out, and 1 μM CAP1 polypeptides (or control buffer) were flowed into the chamber and depolymerization was immediately monitored. *C*, representative field of view from mf-TIRF assay showing filaments with their pointed ends anchored to spectrin-actin (*left*) and their growing barbed ends (*right*). Fluorescent TransFluoSpheres included in all reactions for drift correction. *D*, representative kymographs of actin filament barbed ends depolymerizing in the presence of 1 μM C-CAP1 or control buffer. *Cyan line*, slope of polymerization during initial filament assembly. *Magenta line*, slope of depolymerization at the barbed end after washing out free actin monomers and introducing C-CAP1 or control buffer. *E*, quantification of barbed end depolymerization rates in the presence and absence of 1 μM CAP1 or C-CAP2. The data (mean ± SD) are from three independent replicates (n = 30 filaments total per condition). One-way ANOVA followed by Tukey’s multiple comparisons test was used to determine significance between indicated conditions (∗∗*p* < 0.001; ∗∗∗*p* < 0.0001). Domains: OD, oligomerization domain; HFD, helical-folded domain; P_1_, polyproline 1; W, Wiskott Aldrich syndrome 2 (WH2) domain; P_2_, polyproline 2; CARP, CAP and RP2 (CARP) domain. BE, barbed end; C, carboxyl terminus; CAP, cyclase-associated protein; C-CAP, C-terminal half of CAP; FL, full-length; mf-TIRF, microfluidics-assisted total internal reflection fluorescence; N, amino terminus; PE pointed end.

Whereas N-CAP forms hexamers that interact with actin filaments, the C-terminal half of CAP (C-CAP) is dimeric and until now has only been shown to interact with actin monomers. C-CAP is composed of a ‘polyproline motif 1 - Wiskott Aldrich syndrome homology domain 2 domain - polyproline motif 2' (PWP) region and a ‘CAP and RP2’ (CARP) domain (Fig. 1*A*). Within the PWP region, the P1 binds profilin, the Wiskott Aldrich syndrome 2 (WH2) domain binds actin, and the P2 binds SH3 domains (56, 57, 58). The WH2 domain binds to ATP-actin and ADP-actin monomers with similar affinity (K_d_ ∼ 1 μM) (57). The CARP domain also binds to actin monomers but exhibits ∼100-fold higher binding affinity for ADP- *versus* ATP-actin monomers (59). As a unit, the WH2 and CARP domains together drive actin monomer recycling, catalyzing the dissociation of cofilin from ADP-actin monomers, promoting nucleotide exchange (ATP for ADP) on G-actin, and releasing profilin-bound ATP-actin monomers for new rounds of actin assembly (49, 53, 57, 59, 60). Indeed, in a closed *in vitro* reconstitution system, CAP is critical for recycling actin monomers and maintaining actin-based motility (61).

Thus, until now, the broad picture that has been painted for CAP is that it uses its N-terminal half to bind F-actin (sides and pointed ends) and promote disassembly, while its C-terminal half is used to recharge actin into ATP- and profilin-bound monomers. In the present study, we investigated whether CAP influences dynamics at the barbed ends of actin filaments and discovered that CAP accelerates barbed end depolymerization in the absence of actin monomers, attenuates barbed end growth in the presence of actin monomers, and displaces formins from growing barbed ends. These functions are mediated by the C-terminal WH2 and CARP domains of CAP. Moreover, we found that CAP directly collaborates with profilin in barbed end depolymerization and that this activity depends on their direct physical association. These findings extend our understanding of the mechanistic roles played by CAP in promoting F-actin turnover to include both ends of filaments.

## Results

### CAP1 and CAP2 promote depolymerization at the barbed ends of actin filaments

Using microfluidics-assisted total internal reflection fluorescence (mf-TIRF) microscopy, we investigated the potential effects of mouse CAP1 and CAP2 (Fig. 1*A*) at actin filament barbed ends. In our experimental set up, we assembled fluorescently labeled actin filaments from spectrin-actin seeds attached to the coverslip in microfluidic chambers (Fig. 1*B*). The resulting filaments were anchored at their pointed ends and grew from their free barbed ends. Due to the constant flow, the filaments remained uniformly aligned (Fig. 1*C*). Once filaments reached an optimal length (average ∼10 μm), we washed out free actin monomers, flowed in proteins or control buffer, and immediately monitored depolymerization (see kymographs, Fig. 1*D*). Depolymerization rates were determined from the slopes of the shortening barbed ends (magenta lines, Fig. 1*D*).

In control buffer, barbed ends depolymerized at a rate of 3.58 ± 0.59 subunits s^−1^ (±SD), similar to previously observed rates (27, 36). However, in the presence of 1 μM full-length (FL) CAP1, the barbed ends depolymerized ∼4-fold faster at a rate of 12.9 ± 2.42 subunits s^−1^ (Fig. 1*E*). Similar effects were observed for 1 μM C-CAP1 (13.06 ± 2.52 subunits s^−1^). However, the rate for 1 μM N-CAP1 was indistinguishable from control buffer (3.88 ± 0.79). These results indicate that the C-terminal half of CAP1 (C-CAP1) is required and sufficient to accelerate barbed end depolymerization.

Because there are two CAP genes in mammals (CAP1 and CAP2), we also asked whether the muscle-specific isoform (CAP2) affects barbed end depolymerization. We were unsuccessful in purifying FL CAP2 but found that C-CAP2 accelerated barbed end depolymerization 2.5- to 3-fold, at a rate of 10.82 ± 1.97 subunits s^−1^ (Fig. 1*E*). Thus, the barbed end depolymerization activity is conserved in both CAP isoforms.

### CAP1 promotes depolymerization at the barbed ends of ADP- and ADP-P_i_ actin filaments

**Figure 2:**
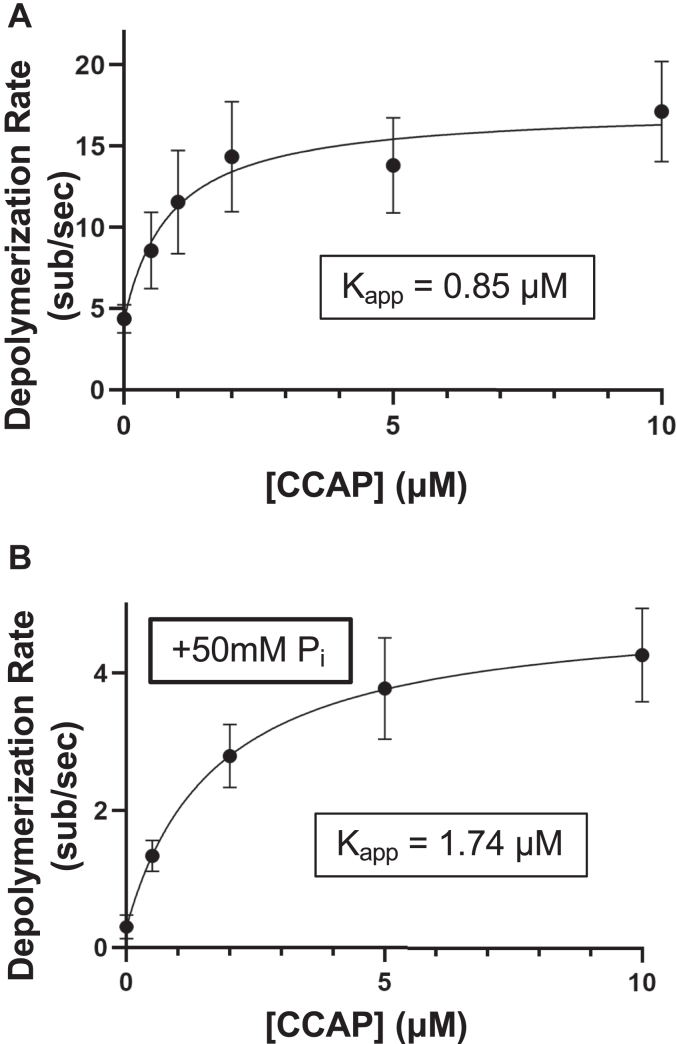
**C-CAP1 promotes depolymerization of ADP-P**_**i**_**actin filaments.***A*, rates of barbed end depolymerization at different concentrations of C-CAP1 in mf-TIRF assays. The data (mean ± SD) are from three independent replicates (n = 30 filaments total per condition). The line is a fit to a hyperbolic binding curve and was used to derive the K_app_ (0.85 μM). *B*, rates of barbed end depolymerization at different concentrations of C-CAP1 in mf-TIRF assays in the presence of 50 mM inorganic phosphate (P_i_). Data (mean ± SD) are from two independent replicates (n = 20 filaments total per condition). The line is a fit to a hyperbolic binding curve and was used to derive the K_app_ (1.74 μM). CAP, cyclase-associated protein; C-CAP, C-terminal half of CAP; mf-TIRF, microfluidics-assisted total internal reflection fluorescence.

To better understand the effects of C-CAP1 at the barbed end, we compared the rates of depolymerization over a range of C-CAP1 concentrations (Fig. 2*A*). C-CAP1 promoted depolymerization in a concentration-dependent manner with a K_app_ of 0.85 μM and a maximal depolymerization rate ∼4-fold faster than control (control, 4.35 subunits s^−1^; 10 μM C-CAP1, 17.12 subunits s^−1^). Thus, at the concentration used in most of our experiments (1 μM C-CAP1), we are at ∼75 to 80% maximal effect. This concentration of CAP1 is well below the 6 to 7 μM CAP1 found in NIH3T3 cells (43, 62).

Previous mf-TIRF studies have shown that aged barbed ends (comprised entirely of ADP-actin) depolymerize at a rate of ∼3 to 10 subunits s^−1^, whereas filaments comprised of ADP-Pi actin depolymerize at least ten times slower at a rate of ∼0.3 subunits s^−1^ (27, 36, 63). In our mf-TIRF experiments above, we polymerized filaments for a period of time and then abruptly (within a 5 s window) washed out actin monomers and flowed in CAP proteins or control buffer and began monitoring depolymerization. Thus, filaments were never intentionally ‘aged’. This was deliberate, as CAP is found at the leading edge actin networks of mammalian cells (43), which turnover in ∼20 to 30 s (39). Therefore, CAP interacts with filaments *in vivo* within seconds of their polymerization, which our experimental design attempts to mimic.

The depolymerization rate in our control reactions was ∼3 to 5 subunits s^−1^, suggesting that the barbed ends of our filaments are comprised primarily of ADP-actin rather than ADP+Pi actin. This observation may be explained by the increased rate of P_i_ release near barbed ends (64, 65). However, this also left open the question of whether CAP promotes the depolymerization of ADP-P_i_ actin. Therefore, we also tested the concentration-dependent effects of C-CAP1 in the presence of 50 mM inorganic phosphate (Fig. 2*B*), which maintains F-actin in the ADP-P_i_ state (66). As expected, the control rate for barbed end depolymerization of ADP-P_i_ actin filaments was about ten times slower (0.30 subunits s^−1^) than the control rate in the absence of inorganic phosphate (36). The presence of C-CAP1 increased the depolymerization rate of ADP-P_i_ filaments by 14-fold at the highest concentration of C-CAP1 tested (10 μM) to a rate of 4.26 subunits s^−1^ and a K_app_ of 1.74 μM. Thus, C-CAP1 accelerates barbed end depolymerization of both ADP- and ADP-P_i_ actin filaments.

### The WH2 and CARP domains of CAP1 mediate barbed end depolymerization

**Figure 3:**
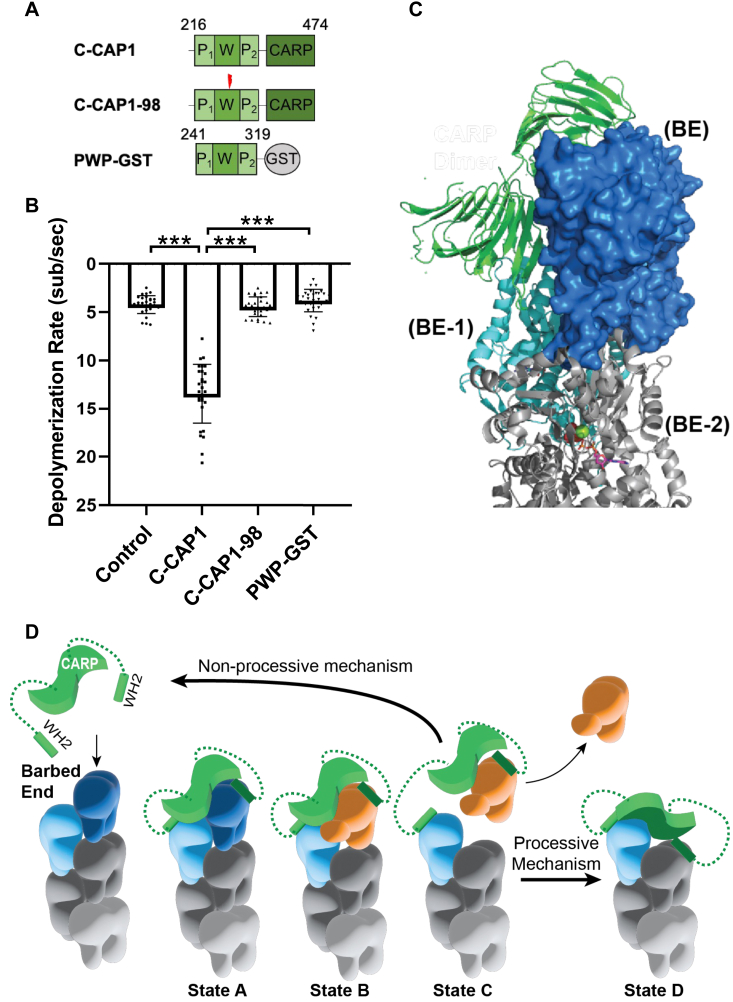
**The barbed end depolymerization activity of CAP requires both its WH2 and CARP domains.***A*, schematics of mouse C-CAP1, C-CAP-98 (^270^LKHV^273^ > AAAA) which disrupts actin binding by the WH2 domain and P_1_WP_2_-GST which is the polyproline motif 1-Wiskott Aldrich syndrome homology domain 2 domain-polyproline motif 2 fragment dimerized by GST. Mutations marked by lightning bolts. *B*, rates of barbed end depolymerization for each construct (1 μM). The data (mean ± SD) are from three independent replicates (n = 30 filaments total per condition). ∗∗∗, one-way ANOVA followed by Tukey’s multiple comparisons test to determine significance between indicated conditions (*p* < 0.0001). *C*, a structural model for the interactions of C-CAP1 with the barbed end (BE) of the actin filament assembled, assembled from a cryo-EM structure of F-actin (PDB #6FHL), and the cocrystal structure of the CAP1 CARP dimer bound to two actin monomers (PDB #6FM2). One actin from the CARP domain-actin cocrystal structure was used to orient the CARP domain on the terminal barbed end actin protomer (BE, in *dark blue* surface representation). The CARP domain is able to fit with only a minor clash against the penultimate actin protomer (BE-1, in *cyan cartoon* representation). *D*, working model for how C-CAP accelerates depolymerization at the BE. Binding of C-CAP to the BE of the filament (state A) involves the two WH2 domains binding to the ultimate and penultimate actin subunits (*dark blue* and *light blue*) and one half of the CARP dimer binding to the ultimate subunit (*dark blue*). Next, interactions of the CARP and/or WH2 domains promote a conformational change in the ultimate actin subunit (*orange*, state B), increasing the rate of dissociation of the ultimate actin subunit from the BE, while the C-CAP dimer remains attached to the BE *via* its WH2 domain (state C). Finally, the original BE actin subunit (*orange*) is released from one half of the CARP dimer. As indicated, if the mechanism is nonprocessive, C-CAP1 then dissociates from the BE and becomes available for a new round of depolymerization. However, if the mechanism is processive, C-CAP1 remains bound to the BE by its WH2 domain after the actin subunit is released, and then the other face of the CARP dimer binds to the new ultimate actin subunit (*light blue*, state D). In the processive mechanism, this cycle would repeat itself until C-CAP1 dissociates from the barbed end. Domains: P_1_, polyproline 1; W, Wiskott Aldrich syndrome homology domain 2; P_2_, polyproline 2; CARP, CAP and RP2 (CARP) domain; GST, Glutathione-S-transferase. CAP, cyclase-associated protein; C-CAP, C-terminal half of CAP; WH2, Wiskott Aldrich syndrome 2.

We next asked whether the actin-binding WH2 and/or CARP domains of mouse C-CAP1 are required for its barbed end depolymerization effects. We started by assessing the contribution of the WH2 domain. Many WH2 domains bind not only to G-actin but also to the barbed ends of actin filaments to regulate their dynamics (31, 67, 68, 69, 70). To test the importance of actin binding by the WH2 domain of CAP1, we used an alanine substitution mutant (C-CAP1-98: ^270-^LKHV/AAAA^-273^) that abolishes WH2 interactions with G-actin (18) (Fig. 3*A*). In contrast to WT C-CAP1, C-CAP1-98 showed no effects on depolymerization (Fig. 3*B*), demonstrating that actin binding by the WH2 domain is critical for CAP’s barbed end depolymerization activity.

These results led us to consider whether the WH2 domain might be the primary actin-binding site in C-CAP1 required for depolymerization activity, while the CARP domain (a dimer) might serve merely to dimerize the WH2 domains. To test this idea, we generated a PWP-GST fusion, with dimeric GST substituting for the CARP domain. However, the PWP-GST fusion failed to promote depolymerization (Fig. 3*B*). Thus, the WH2 domains in the dimer are required but not sufficient for accelerating barbed end depolymerization. This result suggests that the CARP domain may contribute directly to regulation at the barbed end.

The CARP domain has been shown to bind monomeric actin (59, 71), but its potential interactions with filament ends has not been investigated. Therefore, we modeled how C-CAP1 might use its WH2 and CARP domains to interact with the barbed end and promote depolymerization, utilizing a cryo-EM structure of F-actin and the cocrystal structure of C-CAP1 bound to G-actin (Fig. 3*C*). The CARP domain dimer can make extensive contacts with subdomains 1 and 3 of the terminal actin subunit (BE) with only minimal steric overlap with the penultimate subunit (BE-1). This overlap could be alleviated by small motions within the actin protomers of the barbed, such as those suggested by molecular dynamics modeling (72). Thus, the CARP domain could be accommodated at the barbed end. Notably, these CARP domain interactions occur on the ‘inside of filament’ actin surface, leaving the outer face of the subdomain 1 to 3 cleft accessible for WH2 binding (73).

From these observations, we have put together a working model for how C-CAP might promote depolymerization at the barbed end (Fig. 3*D*). In this model, the C-CAP dimer binds to the barbed-end terminal actin subunits using its WH2 domains, while its CARP domain binds to the terminal actin subunit. Once the terminal actin is bound, the D-loop moves to mimic the conformation found in the cocrystal structure. This weakens the contacts of the terminal actin subunit with the filament, promoting release of the terminal subunit from the barbed end. Next, the released actin subunit dissociates from the CARP domain. If the WH2 domain of C-CAP is engaged with the penultimate actin subunit (B-1), the C-CAP dimer stays engaged and can bind the newly terminal actin subunit with the CARP domain, presumably using the opposite face of the CARP dimer. Our model is agnostic as to whether C-CAP drives barbed end depolymerization with any processivity.

### CAP1 collaborates directly with profilin in promoting barbed end depolymerization

**Figure 4:**
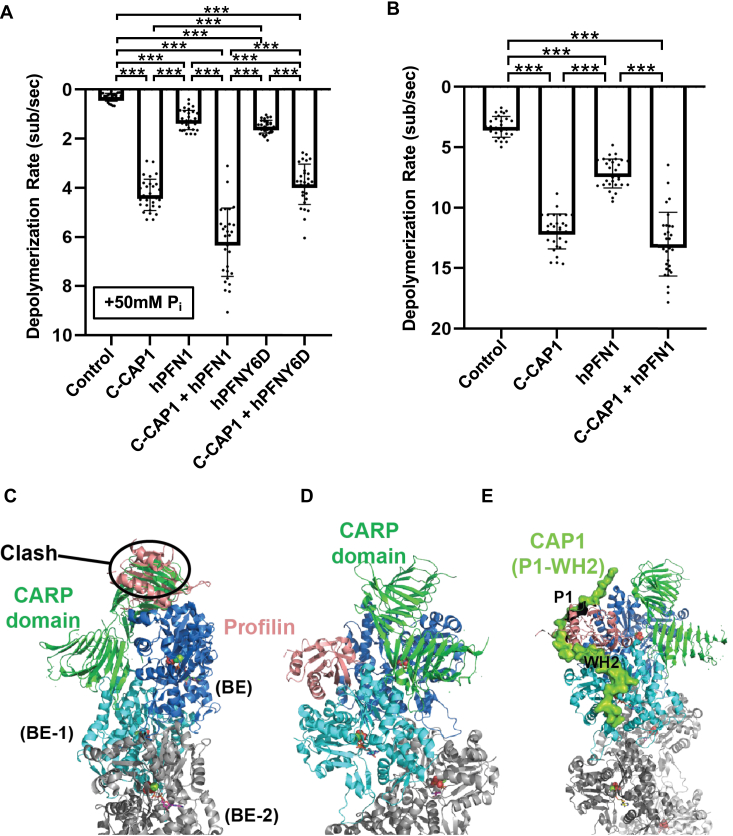
**CAP and profilin directly collaborate in promoting barbed end depolymerization.***A*, rates of barbed end depolymerization in the presence of 10 μM C-CAP1 and 10 μM WT PFN1 or mutant PFNY6D in mf-TIRF assays; all reactions include 50 mM inorganic phosphate (P_i_). The data (mean ± SD) are from three independent replicates (n = 30 filaments total per condition) ∗∗∗, one-way ANOVA followed by Tukey’s multiple comparisons test to determine significance between indicated conditions (*p* < 0.0001). *B*, rates of barbed end depolymerization in the presence of 1 μM C-CAP1 and 10 μM human profilin (PFN1). The data (mean ± SD) are from three independent replicates (n = 30 filaments total per condition). ∗∗∗, one-way ANOVA followed by Tukey’s multiple comparisons test to determine significance between indicated conditions (*p* < 0.0001). *C*, to produce the working models shown in panels *C*–*E*, we docked the CARP domain as in the figure and used a profilin-actin cocrystal structure (PDB ID#: 2BTF) to position profilin on the actin filament model, aligning the actin from the cocrystal structure to the terminal actin (BE). The first model (panel *C*) shows that profilin and the CARP domain cannot both bind to the ultimate actin subunit (BE), as they have a steric overlap that includes most of the volume of profilin. *D*, in this model, profilin binds to the penultimate actin protomer (BE-1) without overlapping with the CARP domain bound to the ultimate subunit (BE). There is a minor overlap of profilin with the ‘hydrophobic plug’ of the terminal actin protomer, but this part of actin should be dynamic when not buried in the filament and could relax out of the minor apparent steric clash. *E*, using AlphaFold2, we generated a working model for how the CAP1 P1-WH2 region may interact with the barbed end, with the P1 motif bound to profilin and the WH2 domain bound to actin. When aligned to the penultimate actin subunit (BE-1), the P1-WH2 region has no steric clashes with profilin, actin, or the CARP domain. CAP, cyclase-associated protein; CARP, CAP and RP2 domain; C-CAP, C-terminal half of CAP; mf-TIRF, microfluidics-assisted total internal reflection fluorescence; WH2, Wiskott Aldrich syndrome 2.

Another cellular factor that promotes barbed end depolymerization is profilin (34). This is intriguing because profilin also binds directly to the PWP region of CAP (Kd = 1.3 μM; (56)), raising the possibility that profilin and CAP could work together in accelerating depolymerization. To test this idea, we compared the effects of mouse C-CAP1 and human profilin (hPFN1), individually and together, on barbed end depolymerization. We included 50 mM inorganic phosphate (P_i_) in the reactions since profilin has been shown to more effectively promote the barbed end depolymerization of ADP-Pi-actin *versus* ADP-actin filaments (34). In these assays, 1 μM C-CAP1 alone showed stronger depolymerization activity than 10 μM hPFN1 alone; however, together they exhibited additive effects (Fig. 4*A*). Since profilin interacts directly with the P1 polyproline tract of CAP (56), we tested a polyproline binding–impaired mutant profilin (PFN1-Y6D) (74). On its own, PFN1-Y6D showed depolymerization activities similar to WT PFN1, as expected. However, PFN1-Y6D effects were not additive with C-CAP1, suggesting that direct CAP–profilin interactions are required for their collaboration in barbed end depolymerization. In reactions lacking free inorganic phosphate (P_i_), PFN1 and C-CAP1 each promoted depolymerization, but their combined effects were not significantly greater than those of C-CAP1 alone (Fig. 4*B*). Thus, PFN1 and C-CAP1 appear to directly collaborate in accelerating the barbed end depolymerization of ADP-Pi-actin filaments but not ADP-actin filaments.

These observations also raise the question of how profilin and C-CAP work together at the barbed end to drive depolymerization and how their direct interactions facilitate these effects. To address this, we structurally modeled profilin and CAP at the barbed end. This revealed that profilin and the CARP domain sterically clash if we model them both bound to the ultimate actin subunit (Fig. 4*C*). In contrast, there is no clash if we model profilin bound to the penultimate subunit and CARP bound to the ultimate subunit (Fig. 4*D*). Further, this second model accommodates binding of the P1 region of C-CAP to profilin (Fig. 4*E*), which may help ‘direct’ profilin to the penultimate actin subunit to enhance depolymerization.

### CAP attenuates filament growth and displaces formins from barbed ends

**Figure 5:**
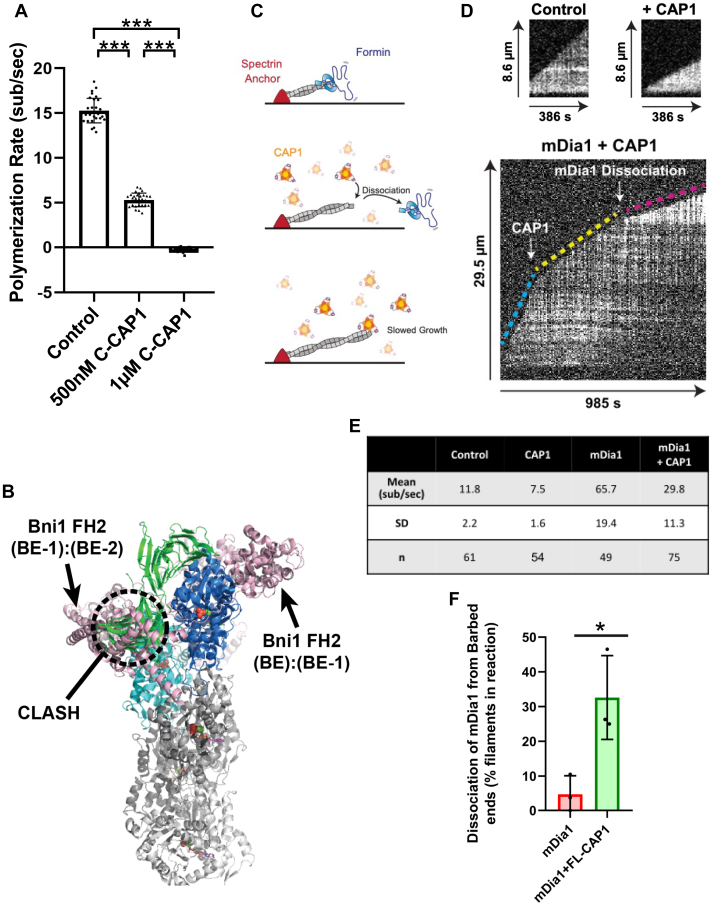
**C-CAP attenuates filament growth and promotes formin dissociation from barbed ends.***A*, barbed end elongation rates in the presence of 2 μM G-actin with or without C-CAP1 (0.5 or 1 μM) in mf-TIRF assays. The data (mean ± SD) are from three independent replicates (n = 30 filaments total per condition). ∗∗∗, one-way ANOVA followed by Tukey’s multiple comparisons test to determine significance between indicated conditions (*p* < 0.0001). *B*, the formin FH2 domain was modeled onto the barbed end using the Bni1-FH2 domain-actin cocrystal structure (PDB ID 1Y64). The actin subunit in the cocrystal was used to orient the FH2 ‘bridge’ domain onto the terminal actin dimer, BE:BE-1, as well as the penultimate actin dimer, BE-1:BE-2 (FH2 bridge units are in *pink* ‘cartoon’ representation). The CARP domain of CAP1 was modeled onto the barbed end as in Figure 4 (same color scheme). In this model, the FH2 bridge at the terminal actin dimer accommodates the CARP domain while the FH2 bridge at the penultimate actin dimer has a substantial clash (indicated). *C*, experimental set up for testing competition between CAP and formin mDia1 at barbed ends. Filaments with free barbed ends were polymerized by exposing coverslip-anchored spectrin-actin seeds briefly to a flow containing 0.5 μM G-actin (10% Alexa-488 labeled) and 2 μM profilin. Twenty millimolar mDia1 was then introduced for 10 s to cap ∼15% of the barbed ends with mDia1 (Top), identified by their faster growth rates. After another 180 s of growth, filaments were exposed to 0.5 μM G-actin (10% Alexa-488 labeled), 2 μM profilin, and 0 or 1 μM full-length (FL) CAP1, and barbed end growth was monitored (*middle* and *bottom*). *D*, representative kymographs of barbed end growth. *Top left*, control reaction containing 0.5 μM G-actin + 2 μM profilin. *Top right*: filament growth in the presence of 1 μM FL-CAP1 (no mDia1). *Bottom*, filament that undergoes three distinct phases of growth, starting with fast growth of mDia1-capped barbed end (*blue dashed line*), followed by slower growth upon addition of 1 μM FL-CAP1 (*yellow dashed line*), and finally slower growth after mDia1 dissociates from the barbed end (*magenta dashed line*). *E*, table showing elongation rates (mean ± SD) of free barbed ends and mDia1-capped ends in the presence and absence of 1 μM FL-CAP1. The data (mean ± SD) are from three independent replicates. Total number of filaments analyzed: n = 61 (control), 54 (+CAP1), 49 (mDia1), 75 (mDia1+FL-CAP1). *F*, quantification of the percent (mean ± SD) of mDia1 dissociation events from barbed ends in the presence and absence of 1 μM FL-CAP1. The data (mean ± SD) are from three independent replicates. Total number of filaments analyzed: n = 111 (mDia1), 108 (+CAP1). ∗Represents statistical significance, *p* < 0.05, between the two groups compared using a Welch’s *t* test. CAP, cyclase-associated protein; C-CAP, C-terminal half of CAP; CARP, CAP and RP2 domain; mf-TIRF, microfluidics-assisted total internal reflection fluorescence.

Finally, we asked whether CAP1 alters barbed end dynamics under growth conditions, *i.e.*, in the presence of actin monomers. For these experiments, we used the same mf-TIRF assay as above except that we included 2 μM G-actin in the flow in (with control buffer or C-CAP1). In control reactions, barbed ends grew at a rate of ∼15 subunits s^−1^ (Fig. 5*A*), as expected for 2 μM G-actin mixtures containing labeled actin (74). Further addition of 0.5 μM C-CAP1 slowed the growth rate to ∼5 subunits s^−1^. Doubling the concentration of C-CAP1 to 1 μM abolished barbed end growth and promoted modest depolymerization (0.26 subunits s^−1^). Importantly, these effects are unlikely to be due to monomer sequestration by C-CAP1, because a maximum of ∼0.8 μM ATP-G-actin should be bound by C-CAP1 under these conditions based on its affinity for ATP-G-actin (K_d_ ∼ 0.38 μM) (75). These observations instead suggest that C-CAP1 interacts with the barbed end of the filament to attenuate growth.

As a second test of CAP1 interactions with barbed ends, we asked whether CAP1 reduces the duration of formin processive attachment at the barbed end, similar to the recently described effects of capping protein and twinfilin (8, 10, 11, 76, 77). A competitive relationship between CAP and formins is predicted by structural modeling, as there is a clash between the CARP domain of CAP and the formin FH2 domain at the penultimate actin subunit of the barbed end (Fig. 5*B*). For these experiments, we grew filaments anchored at their pointed ends by spectrin-actin seeds, then pulsed in formin mDia1 (FH1-FH2-C domain) to cap a small fraction of the barbed ends in reactions (identified by their faster elongation rates) (Fig. 5*C*). Next, we flowed in G-actin and profilin ±1 μM FL CAP1, and after 15 min, we measured the percentage of filaments that had lost mDia1. In kymographs, each phase of barbed end growth is evident. This starts with accelerated growth by mDia1 (blue line, Fig. 5, *D* and *E*). Next, there is a reduced rate of mDia1-capped growth upon addition of CAP1 (yellow line, Fig. 5, *D* and *E*), which may be due to actin monomer sequestration by CAP1 and/or CAP1 binding to profilin which would slow the formin. Finally, there is an even slower rate of growth when mDia1 dissociates from the barbed end (magenta line, Fig. 5, *D* and *E*). Note, the fluorescence intensity of the actin filaments abruptly increases at this stage, which provides an indication of formin dissociation. Previous TIRF studies have shown that filaments polymerized by formins appear much ‘dimmer’ compared to control filaments with free barbed ends (74, 78). This effect is attributed to the bias of profilin in forming complexes (used by formins) with unlabeled actin compared to labeled actin. Quantification of the data from these experiments revealed that the presence of CAP1 markedly increases the frequency of mDia1 dissociation from barbed ends (Fig. 5*F*). Thus, CAP1 not only attenuates growth at free barbed ends but also displaces formins to limit growth.

## Discussion

### CAP is a modular protein with multiple mechanistic roles in driving actin turnover

**Figure 6:**
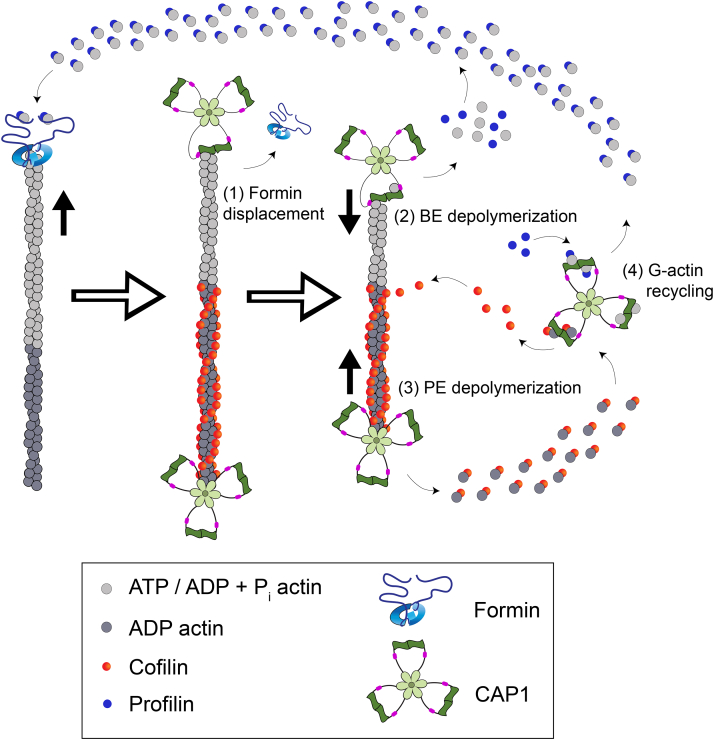
**Model summarizing new and established roles of CAP in promoting actin turnover.** In this study, we have established that (1) CAP (*green*) promotes the displacement of formins (*blue*) from growing barbed ends and (2) that C-terminal domains of CAP (WH2 and CARP) interact with the barbed end (BE) to attenuate growth and promote depolymerization. Further, we have shown that CAP directly collaborates with profilin in promoting BE depolymerization through profilin interactions with the proline-rich P1 region of CAP. Previously, we and others established that (3) the N-terminal HFD domains of CAP interact with the pointed ends (PEs) of actin filaments where they synergize with cofilin (*red circles*) to promote PE depolymerization (54, 55), and (4) C-terminal WH2 and CARP domains of CAP recycle actin monomers, by binding to ADP-G-actin with high affinity, displacing cofilin, catalyzing nucleotide exchange (ATP for ADP) on G-actin, and handing off ATP-G-actin to profilin for new rounds of assembly (18, 49, 53, 57, 59, 60, 71). For simplicity, the ability of N-CAP to bind the sides of actin filaments (using its HFD domains) and enhance cofilin-dependent severing (18, 50, 79, 106) is not depicted in this cartoon but is likely to also contribute to F-actin disassembly. Because cellular concentrations of CAP are relatively high (6–7 μM; (43, 62)), we expect that there are sufficient CAP levels for most of these functions to be occurring simultaneously in cells. CAP, cyclase-associated protein; CARP, CAP and RP2 domain; HFD, helical folded domain; N-CAP, N-terminal half of CAP; WH2, Wiskott Aldrich syndrome 2.

Our findings have revealed that CAP interacts with the barbed ends of actin filaments to attenuate growth, displace formins, and accelerate depolymerization (of ADP-Pi and ADP-actin). These interactions are mediated by C-CAP, which is in striking contrast to the pointed end depolymerization effects of CAP mediated by its N-terminal half (N-CAP) (54, 55). Thus, CAP is a modular protein that uses distinct domains and mechanisms to interact with each end of the filament to promote turnover. Putting these new results together with those of previous studies, it becomes clear that CAP performs multiple mechanistic roles in promoting actin turnover (Fig. 6). N-CAP hexamers use their actin-binding HFD domains to interact with pointed ends of filaments and accelerate depolymerization (54, 55). Although not depicted in Figure 6, N-CAP also uses its HFD domains to bind the sides of filaments and enhance cofilin-mediated severing (18, 50, 79). In contrast, C-CAP dimers use their WH2 and CARP domains (C-CAP) to interact with barbed ends and attenuate growth, displace formins, and promote depolymerization. Moreover, these domains in C-CAP catalyze recharging of actin monomers for new rounds of assembly. This modular view of CAP is supported by genetic observations, as the functions *in vivo* of yeast CAP depend on both its N- and C-terminal halves (50, 53, 57, 59, 80). Moreover, expression of the two halves of yeast CAP *in trans* can rescue a deletion of CAP *in vivo*, and similar to FL CAP, the two halves of CAP *in trans* enhance actin comet tail turnover and bead motility *in vitro* (81).

### Structural basis for CAP effects at the barbed end

Our data show that both the WH2 and CARP domains of C-CAP are required for its ability to induce depolymerization at the barbed end. Based on these findings, we have generated a working model for how C-CAP promotes barbed end depolymerization (Fig. 4*C*). In this model, we assume that the WH2 domain of CAP uses its amphipathic helix to bind the hydrophobic pocket between subdomains I and III in actin, similar to other WH2 domains (73). Using the crystal structure of the CARP dimer bound to G-actin (71), we modeled CARP interactions with the barbed end (Fig. 4*A*). How the interactions of its WH2 and CARP domains with the barbed end increase the rate of depolymerization is not yet clear but may involve weakening of actin–actin interactions to promote subunit dissociation. In addition, we do not yet know how long a CAP dimer stays bound at the barbed end or how many actin subunits are removed with each binding event, but based on the concentration-dependence of CAP’s effects (Fig. 2), it is likely to be transient.

Our structural modeling also helps explain how C-CAP displaces formins from barbed ends. We propose that interactions of the WH2 domain with the penultimate actin subunit at the barbed end allow the C-CAP dimer to ‘get one foot in the door’, while the formin FH2 dimer is stair-stepping and only half-bound. This mechanism is similar to how capping protein uses its actin-binding tentacles (which bind the same sites on actin as WH2 domains) to displace formins and WH2 domains of WAVE from barbed ends (8, 11, 31). We also observed a steric clash at the penultimate subunit between the CARP domain and the formin FH2 domain (Fig. 5*B*), which possibly contributes to formin displacement.

### A collaboration between CAP and profilin at the barbed end

One of the earliest genetic observations for CAP was that a deletion of the C-terminal actin-binding domain of yeast CAP caused defects in cell growth and actin organization which were partially rescued by profilin overexpression (82, 83). A later study showed that profilin binds directly to the ‘P1’ polyproline motif of CAP (56). However, it has remained enigmatic what mechanistic role is supported by direct interactions between profilin and CAP. Here, we showed that CAP and profilin collaborate in accelerating barbed end depolymerization and that this effect depends on their direct interaction. These observations offer a potential explanation for the genetic observations made over 30 years prior and warrant further investigation into possible CAP-profilin collaborations *in vivo* and how they influence cellular actin network turnover.

### Roles for CAP2 in regulating barbed end dynamics in muscle cells

CAP2 is expressed primarily in skeletal and heart muscle tissue, and a knockout of CAP2 in mice leads to defects in muscle architecture and function accompanied by severe dilated cardiomyopathy and muscle weakness (84, 85, 86, 87). A recent study from Gregorio *et al.* (88) showed that CAP2 associates with a dynamic subset of thin filaments in cardiomyocytes. Further, this study showed that knocking down CAP2 altered dynamics at both the pointed ends and barbed ends of the thin filaments. Specifically, after CAP2 depletion, they observed abnormally fast recovery of GFP-actin at the barbed ends. The new activities we report here for CAP1 and CAP2 at barbed ends could explain these *in vivo* observations.

### Broad view: complex molecular interplay at barbed ends

The activities we have described here for CAP at barbed ends contribute to an emerging view of barbed ends as platforms for rich molecular interplay that tunes actin filament dynamics. Formins, Ena/VASP, and some WAVE proteins associate with barbed ends and accelerate filament growth in a profilin-dependent manner. Capping protein and formins displace each other from barbed ends through an associative competition mechanism (8, 11). Capping protein also uses its tentacles to compete with and displace WH2 domains of WASP/WAVE family proteins (31). Formin-binding partners like Spire and IQGAP1 promote formin recruitment and turnover at barbed ends (33, 76). Twinfilin promotes formin displacement from barbed ends and promotes depolymerization of ADP-Pi-actin (10, 36) but also binds to capping protein using its capping protein interaction motif to antagonize other capping protein interaction–containing ligands like CARMIL (89). Given these diverse mechanisms, along with others yet-to-be-discovered, it seems likely that the molecular composition at barbed ends in cells changes rapidly and can be highly complex. The ability of so many different barbed-end associated factors to join each other at actin filament ends, compete and/or cooperate with each other, and produce complex polymer behavior is reminiscent of the rich molecular interplay of +Tip factors that control dynamics at microtubule ends (90, 91).

## Experimental procedures

### Plasmid construction

The pET28a-CAP2 (pBG2189) plasmid for expressing C-CAP2 (219–476) was produced by Gibson Assembly cloning. The pET-28aV2 plasmid for expressing CAP1-PWP (241–319) fused to PreScission Protease cleavable GST, CAP1-PWP-GST, was generated using Gibson Assembly cloning with a pET28aV2 backbone and three overlapping inserts: PWP, PreScission Protease site, and GST. All plasmids were verified by Sanger sequencing. Plasmids for expressing FL CAP1, the N- and C-terminal halves of CAP1, the C-CAP1-98 mutant, profilin, profilin-Y6D mutant, and mDia1 (FH1-FH2-C) were previously described (18, 74, 92).

### Protein purification

Rabbit skeletal muscle actin was purified from acetone powder (93), generated from frozen ground hind leg muscle tissue of young rabbits (Pel-Freez Biologicals). Lyophilized acetone powder stored at −80 °C was mechanically sheared in a coffee grinder, resuspended in G-buffer (5 mM Tris–HCl, pH 8.0, 0.2 mM ATP, 0.5 mM DTT, 0.1 mM CaCl_2_), and then cleared by centrifugation for 20 min at 50,000*g*, 4 °C. The supernatant was filtered through Grade 1 Whatman paper, then the actin was polymerized by the addition of 2 mM MgCl_2_ and 50 mM NaCl to the filtrate and overnight incubation at 4 °C with slow stirring. The next morning, NaCl powder was added to a final of 0.6 M, and stirring was continued for another 30 min at 4 °C. F-actin was pelleted by centrifugation for 150 min at 120,000*g*, 4 °C. The pellet was solubilized by dounce homogenization and dialyzed against 1 l of G-buffer at 4 °C (three consecutive times at 12–18 h intervals). Monomeric actin was then precleared for 30 min at 435,000*g*, 4 °C, and loaded onto a Sephacryl S-200 (16/60) gel-filtration column (Cytiva). Peak fractions containing actin were stored at 4 °C.

For the fluorescently labeled actin used in TIRF assays, actin was labeled on surface-exposed primary amines as previously described (94). Briefly, G-actin was polymerized by dialyzing overnight against modified F-buffer (20 mM PIPES pH 6.9, 0.2 mM CaCl_2,_ 0.2 mM ATP, 100 mM KCl). Then the F-actin was incubated for 2 h at room temperature with a five-fold molar excess of Alexa-488 NHS ester dye (Life Technologies). F-actin was then pelleted by centrifugation at 450,000*g* for 40 min at room temperature. The pellet was resuspended in G-buffer, homogenized with a dounce, and incubated on ice for 2 h to depolymerize filaments. Actin was then repolymerized on ice for 1 h after adding KCl and MgCl_2_ (final concentration of 100 mM and 1 mM respectively). F-actin was pelleted by centrifugation for 40 min at 450,000*g* at 4 °C. The pellet was homogenized with a dounce and dialyzed overnight at 4 °C against 1 l of G-buffer. Next, the solution was centrifuged for 40 min at 450,000*g* at 4 °C, and the supernatant was collected. The concentration and labeling efficiency was determined by measuring the absorbance at 280 nm and 495 nm, using these molar extinction coefficients: ε_280_ actin = 45,840 M^–1^ cm^–1^, ε_495_ Alexa-488 = 71,000 M^–1^ cm^–1^, and ε_280_ AF488 = 7810 M^–1^ cm^–1^.

Human profilin-1 (PFN1) was expressed in *Escherichia coli* BL21 DE3 by growing cells to log phase at 37 °C in Terrific Broth (TB) media and inducing expression with 1 mM IPTG at 37 °C for 3 h. Cells were harvested by centrifugation and pellets were stored at −80 °C. Cell pellets were resuspended in lysis buffer (50 mM Tris–HCl, pH 8.0, 1 mM EDTA, 0.2% Triton X-100, lysozyme,1 mM PMSF, and protease inhibitor cocktail: 0.5 μM each of pepstatin A, antipain, leupeptin, aprotinin, and chymostatin) and kept on ice for 30 min. Lysates were cleared for 30 min at 272,000*g* at 4 °C, and the supernatant was collected and fractionated on a HiTrap Q column (Cytiva) equilibrated in 20 mM Tris, pH 8.0, and 50 mM NaCl and eluted with a salt gradient (0–1 M NaCl and 20 mM Tris, pH 8.0). Peak fractions were concentrated and then purified further on a Superdex 75 (10/300) (Cytiva) column equilibrated in 20 mM Tris–HCl pH 8.0 and 50 mM NaCl. Peak fractions were pooled, snap frozen in aliquots, and stored at −80 °C.

Human PFN1 mutant (Y6D) was purified with slight modifications as described in (95). PFN1(Y6D) was expressed in *E. coli* BL21(DE3) pRARE by growing cells to log phase in TB and inducing expression with 1 mM IPTG overnight at 18 °C. Cells were harvested by centrifugation and pellets stored at −80 °C. Cell pellets were resuspended in lysis buffer (50 mM Tris–HCl, pH 7.5, 20 μg/ml DNase, 1 mM PMSF, and a standard mixture of protease inhibitors) and then lysed in a microfluidizer at 18,000 psi. Lysates were cleared by centrifugation for 20 min at 100,000*g*. Precleared lysate was passed over a HiTrap Q column (Cytiva) equilibrated in lysis buffer and flowthrough was collected containing PFNY6D. This flowthrough was applied to a Superdex 75 (10/300) (Cytiva) equilibrated in storage buffer (50 mM Tris–HCl, pH 8, 50 mM KCl, 1 mM DTT). Peak fractions were pooled, concentrated, and snap frozen to be stored at −80 °C.

FL MBP-CAP1 was expressed in *E. coli* BL21(DE3) pRARE by growing cells to log phase in TB and inducing expression with 1 mM IPTG overnight at 18 °C. Cells were harvested by centrifugation and pellets stored at −80 °C. Cell pellets were resuspended in lysis buffer (50 mM Tris–HCl, pH 8.0, 200 mM NaCl, 1 mM EDTA, pH 8.0, 1 mM DTT, 20 μg/ml DNase, 1 mM PMSF, and a standard mixture of protease inhibitors) and then lysed in a microfluidizer at 18,000 psi. Lysates were cleared by centrifugation for 20 min at 100,000*g*. Precleared lysates were mixed with 1 ml amylose resin (New England BioLabs) and incubated for 1 h rotating at 4 °C. Beads were then washed with ten column volumes of binding buffer (50 mM Tris–HCl, pH 8.0, 200 mM NaCl, 1 mM EDTA, pH 8.0, 1 mM DTT). Proteins were eluted in elution buffer (binding buffer + 50 mM Maltose), concentrated, cleared by low-speed centrifugation, and gel filtered on a Superdex 200 Increase (10/300 GL) column (Cytiva) equilibrated in storage buffer (20 mM Tris–HCL, pH 8.0, 200 mM NaCl, 1 mM DTT). Peak fractions were pooled, concentrated, and snap frozen to be stored at −80 °C.

C-terminal CAP polypeptides (C-CAP1-6His, C-CAP1-98-6His mutant, and C-CAP2-6His) were purified as described (18). Protein was expressed in *E. coli* BL21(DE3) pRARE by growing cells to log phase in TB and inducing expression with 1 mM IPTG overnight at 18 °C. Cells were harvested by centrifugation and pellets stored at −80 °C. Cell pellets were resuspended in lysis buffer (20 mM potassium-phosphate, pH 7.4, 300 mM NaCl, 10 mM imidazole, 20 μg/ml DNase, 1 mM PMSF, and a standard mixture of protease inhibitors) and then lysed in a microfluidizer at 18,000 psi. Lysates were cleared by centrifugation for 20 min at 100,000*g*. Precleared lysates were mixed with 1 ml Ni-NTA-agarose beads (Qiagen) and incubated for 2 h rotating at 4 °C. Beads were then washed with ten column volumes of wash buffer (20 mM potassium phosphate, pH 7.4, 300 mM NaCl, 50 mM imidazole). Proteins were eluted in elution buffer (20 mM potassium phosphate, pH 7.4, 300 mM NaCl, 250 mM imidazole), concentrated, cleared by low-speed centrifugation, and gel filtered on a Superdex 200 increase (10/300 GL) column (Cytiva) equilibrated in storage buffer (20 mM Tris–HCl, pH 8.0, 50 mM NaCl, 1 mM DTT). Peak fractions were pooled, concentrated, and snap frozen to be stored at −80 °C.

N-CAP1-6His was expressed and purified as described (96). Transformed *E. coli* BL21(DE3) pRARE cells were grown and induced as above for C-CAP1. Cells were resuspended and lysed by sonication in lysis buffer (20 mM phosphate buffer pH 7.4, 300 mM NaCl, 1 mM DTT, 10 mM imidazole, and a standard mixture of protease inhibitors). Lysates were cleared by centrifugation for 20 min at 100,000*g*. Precleared lysates were incubated with 1 ml Ni-NTA-agarose beads (Qiagen) for 90 min rotating at 4 °C. Beads were then washed with ten column volumes of lysis buffer supplemented with 50 mM imidazole. Proteins were eluted with five column volumes of lysis buffer supplemented with 250 mM imidazole, concentrated, cleared by low-speed centrifugation, and purified further on a Superose 6 (10/300) gel filtration column (Cytiva) equilibrated in storage buffer (20 mM Tris–HCl pH 8.0, 100 mM NaCl, 1 mM DTT). Peak fractions were pooled, concentrated, and snap frozen to be stored at −80 °C.

6His-PWP-GST was expressed as above for C-CAP1. Cell pellets were resuspended in lysis buffer (50 mM Tris–HCl, pH 8, 150 mM NaCl, 1 mM DTT, 20 μg/ml DNase, 1 mM PMSF, and a standard mixture of protease inhibitors) and then lysed using a microfluidizer at 18,000 psi. Lysates were cleared by centrifugation for 20 min at 100,000*g*. The precleared lysate was mixed with 1 ml glutathione-agarose beads (Thermo Fisher Scientific) and incubated for 1 h rotating at 4 °C. Beads were then washed ten times with GST wash buffer (50 mM Tris–HCl, pH 8, 150 mM NaCl, 1 mM DTT) and eluted in 4 ml of GST elution buffer (GST wash buffer supplemented with 10 mM reduced glutathione (Sigma)). Four milliliters of elution was mixed with 46 ml of Ni binding buffer (20 mM potassium phosphate, pH 7.4, 300 mM NaCl, 10 mM imidazole), 1 ml Ni-NTA-agarose beads (Qiagen) and incubated for 1 h rotating at 4 °C. Beads were then washed with ten column volumes of wash buffer (20 mM potassium phosphate, pH 7.4, 300 mM NaCl, 50 mM imidazole). Proteins were eluted in elution buffer (20 mM potassium phosphate, pH 7.4, 300 mM NaCl, 250 mM imidazole). Peak fractions were pooled, buffer exchanged into storage buffer (20 mM Tris–HCL, pH 8.0, 50 mM NaCl, 1 mM DTT), concentrated, snap frozen, and stored at −80 °C.

The active FH1-FH2-C fragment of mouse formin mDia1 (residues 553–1255) was expressed from a high copy plasmid in *Saccharomyces cerevisiae* and purified as described (97). Briefly, 2 l of cells were grown at 25 °C to A_600_ = 0.8 in selective medium containing 2% raffinose. Galactose (2% final) was added to induce expression for 12 to 16 h. Yeast cells were harvested by centrifugation and resuspended in a 1:3 (v/w) ratio of water, mechanically lysed in a coffee grinder with liquid N_2_, and stored at −80 °C. Frozen yeast powder was thawed in a 1:4 (v/w) ratio of 20 mM imidazole pH 8.0, 1.5× PBS buffer (pH 7.4), 0.5% NP-40, 0.2% Thesit, 1 mM DTT, and protease inhibitors. Next, the lysate was clarified by centrifugation at 150,000*g* for 80 min at 4 °C and then incubated with Ni-NTA agarose beads (Qiagen) for 1.5 h at 4 °C. The beads were washed three times with 1× PBS buffer, and the protein was eluted with 0.5 ml of 300 mM imidazole pH 8.0, 50 mM Tris–HCl pH-8.0, 100 mM NaCl, 1 mM DTT, 5% glycerol, then purified further on a Superose 6 (10/300) gel filtration column (Cytiva) equilibrated in storage buffer (20 mM Tris–HCl pH 8.0, 100 mM NaCl, 1 mM DTT). Peak fractions were pooled, concentrated, aliquoted, snap frozen, and stored at −80 °C.

Spectrin-actin seeds, for mfTIRF, were purified from blood as described in (98, 99). Briefly, 50 ml of packed human red blood cells (Research Blood Components) were washed with three times with 25 ml of ice-cold buffer A (5 mM sodium phosphate, pH 7.7, 150 mM NaCl, and 1 mM EDTA), each time centrifuging for 15 min at 2000*g* at 4 °C and discarding the supernatant. To lyse cells, the cell pellet was resuspended in 700 ml (approximately ten times the volume of washed cells) of ice-cold lysis buffer (5 mM sodium phosphate, pH 7.7 and 1 mM PMSF) and incubated for 40 min while stirring at 4 °C. The lysate was centrifuged for 15 min at 45,000*g* at 4 °C. The cloudy and viscous pellets were resuspended in wash buffer B (5 mM sodium phosphate, pH 7.7 and 0.1 mM PMSF), final volume 360 ml and homogenized by pipetting. Next, the mixture was centrifuged for 15 min at 45,000*g* at 4 °C. The pellets were resuspended in a total volume of 180 ml of wash buffer B and homogenized as above, then centrifuged as above. This process was repeated once more. Pellets are translucent at this stage. Next, the Spectrin-actin was extracted by resuspending each pellet in 5 ml of extraction buffer (0.3 mM sodium phosphate, pH 7.6 and 0.1 mM PMSF), combining the contents into one tube, adjusting the volume to 60 ml with the same buffer, and centrifuging for 30 min at 60,000*g* at 4 °C, repeated once. The final pellet was resuspended in an equal volume of extraction buffer and gently vortexed, then incubated for 40 min in a water bath at 37 °C while manually inverting the tubes every ∼10 min. Finally, the sample was precleared for 30 min at 450,000*g* at 4 °C. DTT (2 mM final) and protease inhibitors were added to the cleared supernatant, and an equal volume of cold glycerol (50% final concentration) was mixed into the solution. Spectrin-actin seeds were aliquoted and stored at −20 °C.

### Molecular modeling

Molecular modeling was performed using the PyMOL Molecular Graphics System, Version 2.4.2 (Schrödinger, LLC) and the following pre-existing structures: PDB# 6FHL actin filament (100), 6FM2 (CAP1:actin complex) (71), PDB# 1Y64 (mDIA FH2-actin complex) (101), and PDB# 2BTF profilin–actin complex (102). ColabFold (103, 104) was used to produce a model of CAP1 P1-WH2 region bound to actin-profilin. The resulting model was evaluated for self consistency across five modeling cycles, reasonable pIDDT scores, similarity to models produced for Srv2 and CAP2, and similarity to a pre-existing VASP poly-PRO-GAB region bound to profilin-actin (PDB# 2PBD) (105). Models were constructed by using the PyMol ‘super’ command to overlay relevant structures, which automatically refines aligned regions to exclude outliers.

### mf-TIRF microscopy

Actin filaments were first assembled in flow cells as described in (99). Glass coverslips (25 × 25 mm; Coring) were first cleaned by sonication in detergent for 60 min, followed by successive sonication in 1 M KOH and 1 M HCl for 30 min each and in 100% ethanol for 60 min. Coverslips were then washed extensively with H_2_O and dried in an N_2_ stream. Cleaned coverslips were coated with a 80% ethanol (pH 2.0) solution containing 10 mg/ml methoxy-PEG–silane MW 2000 and 4 μg/ml biotin-PEG-silane MW 3400 (Laysan Bio) and incubated until used at 70 °C. A polydimethylsiloxane mold with three inlets and one outlet was mechanically clamped onto a PEG-silane–coated coverslip. The chamber was then connected to a Maesflo microfluidic flow-control system (Fluigent), rinsed with TIRF buffer (50 mM imidazole pH 7.4, 50 mM KCl, 1 mM MgCl_2_, 1 mM EGTA, 0.2 mM ATP, 10 mM DTT, 15 mM glucose) + TransFluoSpheres (Thermo Fisher Scientific) used in our analysis for drift correction. Next, spectrin-actin seeds in TIRF buffer were passively absorbed to the coverslip for 10 min followed by incubation for 10 min with 1% bovine serum albumin, then washed with TIRF buffer. Next, to polymerize actin filaments with free barbed ends, 2 μM G-actin (10% Alexa-488 labeled) in TIRF buffer was introduced. Once filaments were grown to the desired length (5–15 μm unless otherwise specified), specific proteins were flowed in, and barbed end depolymerization was monitored. For experiments containing 50 mM inorganic phosphate (P_i_), a modified TIRF buffer was used containing 50 mM PO_4_ instead of KCl (50 mM imidazole pH 7.4, 50 mM PO_4_ (from a mixture of KH_2_PO4 and K_2_HPO4), 1 mM MgCl_2_, 1 mM EGTA, 0.2 mM ATP, 10 mM DTT, 15 mM glucose).

### Image acquisition and analysis

Time-lapse TIRF imaging was performed on a Nikon-Ti2000 inverted microscope equipped with a 150-mW argon laser (Melles Griot), a 60× TIRF-objective with a numerical aperture of 1.49 (Nikon Instruments), and an electron-multiplying charge-coupled device camera (Andor Ixon). One pixel was equivalent to 143 × 143 nm. Focus was maintained by the Perfect Focus system (Nikon Instruments). Images were acquired every 5 s and exposed for 100 ms using imaging software Elements (Nikon Instruments). Images were analyzed in Fiji (National Institutes of Health; https://fiji.sc). Drift correction was performed using coverslip-anchored streptavidin-functionalized TransFluoSpheres (Thermo Fisher Scientific) with the StackReg and Image Stabilizer plugins in ImageJ (https://fiji.sc). Background subtraction was conducted using the rolling ball background subtraction algorithm (ball radius, five pixels). Rates of barbed end depolymerization and polymerization were determined by generating kymographs (FIJI kymograph plug-in) from individual filaments. The kymograph slope was used to calculate barbed end depolymerization/polymerization rate (assuming one actin subunit contributes 2.7 nm to filament length). To measure the number of mDia (FH1-C) dissociation events from barbed ends, the fast-growing actin filaments in the field of view (growing at a rate of >30 subunits/s) were monitored over a 15 min window, and formin dissociation events were scored when growth abruptly slowed (to <14 subunits/s).

Data was plotted and analyzed for statistical significance using GraphPad Prism 8.0 (https://www.graphpad.com). To determine statistical significance, we used one-way ANOVA followed by Tukey’s multiple comparisons test to determine significance between indicated conditions in Figures 1*E*, 3*D*, 5, *A* and *B*, and 6*A*. To determine the significance between the two groups in Figure 6*E*, a Welch’s *t* test was performed. The results for all statistical comparisons can be found in Supporting Information 1. Hyperbolic-binding curves in Figure 2, *A* and *B* were fit using the equation, $Y=B_{0}+\left(\frac{\left(B_{\max}-B_{0}\right)X}{\left(K_{app}+X\right)}\right)$, where B_0_ is the rate of depolymerization in the absence of C-CAP1, B_max_ is the rate of depolymerization at a saturating concentration of C-CAP1, and K_app_ is the rate of depolymerization at the concentration of C-CAP1 that produces half-maximal effects.

## Data availability

All data reported in this paper will be shared by the lead contact upon request.

## Supporting information

This article contains supporting information.

## Conflict of interest

The authors declare that they have no conflicts of interest with the contents of this article.
